# Epidemiological, clinical and prognosis aspects of acute generalized peritonitis in South-Kivu Province: descriptive observational study of 278 cases

**DOI:** 10.11604/pamj.2024.47.1.38288

**Published:** 2024-01-02

**Authors:** Jean Paul Buhendwa Cikwanine, Désiré Munyali Alumeti, Kivukuto John Mutendela, Prisca Kavira Ise-Somo, Jonathan Tunangoya Yoyu, Patrick Musimwa Ciza, Maroyi Raha, Willy Arung Kalau

**Affiliations:** 1 Department of Surgery, Evangelical University in Africa Panzi Hospital, Bukavu, Democratic Republic of Congo,; 2 Médecins d´Afrique, Coordination-Europe, Savigny Sur Orge, France,; 3 Department of Medical Works and Research, Progressive Medical Systems, Goma, Democratic Republic of Congo,; 4 Department of Surgery, Ciriri Rau Hospital, Bukavu Democratic Republic of Congo,; 5 Department of Surgery, University of Lubumbashi, Lubumbashi, Democratic Republic of Congo

**Keywords:** Acute generalized peritonitis, epidemiological, prognosis, Democratic Republic of Congo

## Abstract

**Introduction:**

the aim was to determine epidemiological, clinical, therapeutic, and prognostic aspects of acute generalized peritonitis (AGP).

**Methods:**

we conducted an observational, cross-sectional and multicentre study over 2 years of 278 cases of acute generalized peritonitis operated in semi-urban and urban hospitals in South-Kivu Province, Democratic Republic of Congo.

**Results:**

the population of this study was young with a mean age was 28.9 ± 16.1 years with extremes of 1.3 years to 80 years with a sex ratio M: F of 0.8. Peritonitis aetiology was dominated by intestinal perforation 132 cases (47.4%), the admission time in 65, 5% was more than 72 hours. Acute abdominal pain was the most reason for consultation in 93.2% of cases, 11.9% of patients were in hypovolemic shock. In 40.6%, the treatment of patients consisted in intestinal resection with terminal anastomosis, or ileostomy in 32.7%. About the outcomes, 32.4% of the patients had a surgical reoperation and 15.8% of the digestive fistulas were reported. The average duration of the hospitalization was 23.4 ± 20.3 days. Morbidity rate was 14.7%.

**Conclusion:**

the AGP remains one of the abdominal emergencies observed in different semi-urban and urban hospitals of the province of South-Kivu, causing some problems of medical and surgical management, starting from the delay of admission, the severity of the symptoms related to the etiology of the intestinal perforation. In all cases, AGP requires a well-executed resuscitation procedure and surgical technique to improve the prognosis and reduce mortality, which seems to be high in this study.

## Introduction

Acute Generalised Peritonitis (AGP) is a severe intra-abdominal condition that presents as an acute abdomen and necessitates emergency medical and surgical intervention [1]. This is an inflammation of the peritoneum, the membrane that lines the inner abdominal wall and surrounds the organs in the abdomen. It can be caused by a spontaneous infection or by the spread of a localized abdominal infection following the perforation of a digestive organ [2]. Acute generalised peritonitis has a wide range of aetiologies, including tympanic ileal perforation, perforated peptic ulcers, perforated appendicitis, and complications of intestinal obstruction. The case-fatality rate for peritonitis varies between 8.4% and 34% [1,3]. In the Democratic Republic of Congo, a study carried out in Butembo showed that acute generalized peritonitis is the second most common cause of abdominal emergencies after appendicitis, and its main etiology is typhoid perforation [4]. Acute generalised peritonitis, particularly due to typhoid perforation, affects mainly poor and vulnerable populations living in unsanitary and overcrowded conditions. These populations are often late in seeking medical care after attempting self-medication due to a lack of knowledge of the symptoms and fear of the high cost of surgery [3]. In the subtropical countries of Africa, the delay in the management of patients with AGP is at the root of a worse prognosis [3,5]. Indeed, the delay in initiating management is thought to harm patient prognosis. To our knowledge, there is limited contemporary literature detailing the aetiological, clinical, and prognostic traits of patients who have undergone surgery for AGP, particularly in the South-Kivu Province. This leads to the following inquiries: What are the epidemiological, clinical, and therapeutic features of patients who have had surgery for AGP? What are the outcomes of surgical management for acute generalized Peritonitis (AGP)? What are the expenses associated with treating AGP? The objective of this multicentre study is to describe sociodemographic, clinical, therapeutic, and prognostic characteristics, as well as costs associated with the management of AGP in semi-urban and urban hospitals within South Kivu Province.

## Methods

**Figure 1:**
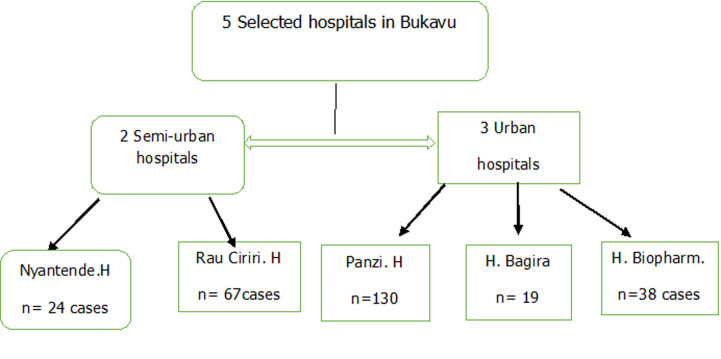
flow chart of selected hospitals and cases

**Study type, location, and duration:** this retrospective, descriptive, observational study was conducted from 1^st^ January to 31^st^ December 2019 in five urban and semi-urban healthcare facilities, selected based on their agreement to participate, attendance, and availability of archived medical records. The semi-urban hospitals of Nyatende and Ciriri, alongside the urban hospitals of Bagira, Panzi, and Biopharm, were included in the study. All these facilities have board-certified surgeons, unlike Nyantende and Bagira, where some surgical procedures are usually performed by residents from the surgical department of Panzi Hospital (Figure 1).

**Study population, data collection and analysis:** our study population consisted of all patients admitted to the surgical department during the study period. The sample was exhaustive. It included all patients admitted for peritonitis whose medical records were available and well-completed. Patients who underwent laparotomy for peritonitis and whose medical records were incomplete were excluded from the study. Epidemiological (age, sex, aetiologies), clinical, and outcome data were collected using an Excel spreadsheet created from medical records, emergency department archives, operating room and intensive care unit registers, and postoperative reports. We considered as AGP all cases of acute abdominal pain with abdominal tenderness or rigidity in which the laparotomy revealed cloudy or purulent fluid throughout the abdominal cavity. Bacteriological and histological analyses were not performed. Tables were used to present the results. Descriptive statistics were performed for sociodemographic, clinical, therapeutic, and prognostic variables, depending on whether they were qualitative or quantitative. Mean or median; standard deviation and range when used to describe quantitative data, and frequency and relative frequency in percentages when used to describe qualitative data. Coding and analyses were performed using Microsoft Excel and epi Info, respectively.

**Ethics and data confidentiality:** approval from the ethics committee of the University of Lubumbashi was obtained under reference UNILU/CEM/166/2018. Confidentiality and anonymity were respected. The data collected were used solely for scientific purposes.

## Results

**Sociodemographic characteristics, etiology, and location of intestinal perforation:** out of 458 cases of gastrointestinal surgical emergencies, a total of 278 (60.7%) cases of AGP were diagnosed in 5. There were 154 females (55.4%) and 124 males (44.6%), with a sex ratio M: F of 0.8. The mean age was 28.9 ± 16.1 years, with extremes ranging from 1.3 to 80 years. The most common age group was 20-40 years. (Table 1). Acute generalised peritonitis´ were secondary in 259 (93.1%) cases and primitive in 19 (6.9%) cases. Iatrogenic origin was found in 24 cases (8.6%). Peritonitis due to intestinal perforation was predominant, accounting for 132 (47.4%) cases, with ileal perforation accounting for 101 (76.5%) cases (Table 2).

**Table 1:** sociodemographic characteristics of patients with acute generalized peritonitis in semi-urban and urban hospitals of South-Kivu Province

Variables	Modalities	Number (n=278)	Percentage (%)
**Age groups (years)**	[0-20]	98	35.3
	(21-40)	130	46.8
	[41-60]	33	11.9
	(61-80)	17	6.1
**Mean age ± SD (Min-Max)**		28.9 ± 16.1 (1.3-80)	
**Gender**	Female	154	55.4
	Male	124	44.6
	Semi-urban	175	62.95
**Area**	Urban	103	37.05

**Table 2:** consultation reasons, clinical signs, ASA aetiologies of acute generalized peritonitis and location of intestinal perforation in semi-urban and urban hospitals of South-Kivu Province

Variables	Numbers	Percentage
**Reference (N=278)**		
Yes	231	83.1
No	47	16.9
**Time elapsed prior to admission in emergency (N=278)**		
≤ 78 hours	96	34.5
>78hours	182	65.5
**Reason for consultation (N=278)**		
Acute abdominal pain	259	93.2
Vomiting	117	42.1
Stoppage of bowel movements and gas	122	43.9
**Clinical signs (N=278)**		
Disturbance of consciousness	19	6.8
Fever	178	64.0
Hypovolemic shock	33	11.9
Anuria	30	10.8
Abdominal tenderness	247	88.8
Contracture	203	73.0
Bloomberg sign	98	35.3
ASA* score (N=278)		
ASA 1 U**	79	28.4
ASA 2 U	177	63.7
ASA 3 U	13	4.7
ASA 4 U	7	2,5
ASA 5 U	2	0.7
**Aetiologies (N=278)**		
Peritonitis due to intestinal perforation	132	47.4
Appendicular peritonitis	35	12.6
Post-operative peritonitis	26	9.4
Iatrogenic peritonitis	24	8.6
Gastric perforation peritonitis	20	7.2
Primitive peritonitis	19	6.8
Post-traumatic peritonitis	12	4.3
Tubo-ovarian or gynaecological peritonitis	10	3.6
**Location of the intestinal perforation (N=132)**		
Ilium	101	76.5
Colic	17	12.9
Ileo-caecal junction	9	6.8
Jejunal	4	3.0
Duodenal	1	0.8

* American society of anaesthesiologists; ** urgent

**Clinical and therapeutic data:** regarding the mode of admission and clinical characteristics, 83.1% (231 cases) were admitted after a few days in a medical structure other than the 5 hospitals involved in this study, and only 16.9% (47 cases) had their first consultation in a reference hospital. About 65% (182) were admitted after more than 72 hours of acute abdominal pain, with a maximum of 14 days before consultation. Acute abdominal pain was the reason for consultation in most patients - 259 (93.2%). Abdominal distension, tenderness or rigidity was the most common physical symptom found in 88.8% (247 cases), followed by fever in 178 (64%). Disturbed consciousness was found in 19 (6.8%) cases. Most patients, 177 (63.7%), were classified as American Society of Anaesthesiologists (ASA) 2 (Table 3). No nutritional data were recorded in any hospitals. After resuscitation, 100% of patients underwent median laparotomy. Abdominal lavage with isotonic saline (NaCl 0.9%) was performed in all patients, although the amount used was not specified in most protocols. For bowel lesions, the surgical procedure performed was bowel resection with terminal anastomosis in 41 (40.6%) cases, or ileostomy in 33 (32.7%) cases, and revascularization of the bowel wound edges followed by suturing in 27 (26.7%) cases. In 123 (44.2%) cases, an abdominal drain was placed in the peritoneal cavity. In 207 (74.5%) cases, the operation was performed either by a resident (Panzi Hospital) or by a general practitioner. Only 71 (25.5%) operations were performed by a registered surgeon. The Ceftriaxone and Metronidazole antibiotic combination was administered in 67.6% of cases, while the Metronidazole-Ciprofloxacin-Ampicillin combination was used in 19.8% of cases.

**Table 3:** patients with generalized acute peritonitis according to treatment received in semi-urban and urban hospitals of South-Kivu Province

Variables	Numbers	Percentage
**Operator (N=278)**		
Resident in surgery or generalist practitioner	207	74.5
Specialist surgeon	71	25.5
**Intervention procedures (N=278)**		
Median laparotomy	278	100
Abdominal cleansing using an isotonic saline solution	278	100
Aspiration du pus	278	100
Abdominal drainage	123	44.2
Bowel resection with terminal anastomosis	41	14.7
Appendicectomy	35	12.6
Ileostomy	33	11.9
Revascularisation of the margins of the bowel wound followed by suturing	27	9.7
Gastrorraphy	20	7.2
Salpingotomy	7	2.5
Hysterectomy	3	1.1
Salpingectomy	1	0.4
**Antibiotics (N=278)**		
Ceftriaxone; Metronidazole	188	67.6
Metronidazole; Ciprofloxacin; Ampicillin	55	19.8
Metronidazole; Gentamicin; Ampicillin	15	5.4
Ceftriaxone; Metronidazole; Ciprofloxacin	6.6	2.4
Sulbactam; Metronidazole	5.5	14.6
Cefotaxime; Metronidazole	4.4	1.6
Cefixime; Metronidazole	2.2	0.8
Doxycycline	1.1	2.9
**Management cost**		
Urban hospital, mean (min; max) United State dollar	800.3 (457-1540)	
Semi-urban hospital, mean (min; max) United State dollar	377.1 (350-544)	

**Surgical care outcomes and prognosis:** the mean length of hospital stay was 23.4 ± 20.3 days, ranging from 1 day (for a deceased patient) to a maximum of 138 days. The most frequent duration of stay was between 11 and 20 days, appearing 116 times (41.7%). A total of 74.1% of patients did not experience any complications during their stay. However, surgical reoperations were necessary for 32.4% of patients, while peritonitis was complicated by a digestive fistula in 15.8%, a parietal infection in 5%, evisceration in 3.2%, any other complications in 1.8% and subphrenic abscess in 1.4%. The overall mortality rate was 14.7% (41 cases), as shown in Table 4. The cost of treatment and hospitalization varied by health facility. The average cost of treatment was 800.3 United States Dollars (USD), ranging from 457 USD to 1540 USD in urban hospitals.

**Table 4:** postoperative outcome of patients operated for acute generalised peritonitis in semi-urban and urban hospitals in South-Kivu Province

Variables		
**Hospital duration, days**		
Mean ±SD (min; max)	23.4 ± 20.3 (1-138)	
	**Numbers**	**Percentage**
(0-10)	55	19.8
(11-20)	116	41.7
(21-30)	46	16.6
> 30	61	21.9
**Post-operative complication**		
Yes	206	74.1
No	72	25.9
**Post-operative complications**		
Digestive fistula	44	15.8
Parietal infection	14	5
Evisceration	9	3.2
Subphrenic abscess	4	1.4
Other complication	5	1.8
**Surgical reoperation**		
Yes	90	32.4
No	188	67.6
**Discharge mode**		
Death	41	14.7
Recovery	237	85,3

## Discussion

This study aimed to determine the sociodemographic, clinical, prognostic, and therapeutic characteristics of acute generalised peritonitis and the costs associated with its management in urban and semi-urban hospitals in the province of South Kivu, in the east of the Democratic Republic of Congo. The results showed that acute generalised peritonitis is a common abdominal digestive emergency in general surgery, with patients presenting late to first-level health facilities where they are referred after consultation with second-level health facilities. The most common aetiology is secondary peritonitis due to bowel perforation, particularly ileal perforation, and management of all cases is often left to non-specialists. Mortality is high and the cost of hospitalisation exceeds known household incomes. The study's findings indicate that acute generalised peritonitis is the most frequent abdominal emergency in surgery, with a rate of 60.2%. It's the second most frequent cause after acute appendicitis in Mali for the year 2020, as reported in reference [6]. This high occurrence rate may be attributed to the prevalence of typhoid fever, lack of water, adequate hand sanitation and gynaecological peritonitis observed in the study. In a similar study conducted in N'Djamena [5], the authors reported a male gender predominance of 86.8% and 13.2% female, an M: F sex ratio of 6.5 and a mean age of 25.87 years (with extremes ranging from 15 to 70 years). This male predominance and young age are also found in other studies [7-9]. In this study, we have a sex ratio F: M of 1.2, with a mean age of 28.9 ±16.1 years, with extremes of 1.3 and 80 years. This discrepancy may be related to the significant number of peritonitis cases of gynaecological origin observed in our series. In our series, we found a significant delay in consultation; 65.5% (182 patients) were admitted more than 72 hours after the onset of acute abdominal pain, with a maximum of 14 days before consultation. This delay in consultation has been reported by other authors [5,7-11]. In sub-Saharan Africa, most patients consult modern medicine only after traditional and herbal treatments have failed. In addition, the lack of financial resources also leads to a delay in hospitalisation until the patient's condition becomes critical.

Disturbance of consciousness (6.8%), hypovolemic shock (11.9%) and ASA 2 classification in 63.7% of cases indicate the severity of the situation in which these patients were admitted and should influence the prognosis. Tobome *et al*. [12] in Benin reported 74.6% of cases classified as ASA3 and Choua [5] 93.7% ASA1 with 8.3% of shock cases. This other discrepancy observed in the ASA classification would depend on the understanding and competence of the assessors, as the scarcity of anaesthetists in certain sub-Saharan African countries is well known. The aetiologies of AGP vary from author to author, often depending on age, sex, lifestyle and diet, and the list is not exhaustive. Three causes seem to be the most common according to the authors: ileal perforation of typhoid origin, appendicular rupture, and gastric perforation [7-9]. We found 132 (47.4%) cases of intestinal perforation with a predominance of ileal perforation in 101 (76.5%). In a study focusing on acute typhoid peritonitis in Kinshasa, the authors confirm that typhoid fever is endemic in this city, as in other developing countries of sub-Saharan Africa, and that it is the most common cause of ileal perforation [13]. Our study results reveal a prevalence rate of 3.6%, encompassing ten instances of gynaecological peritonitis. As for Mali, a previously conducted study has reported a singular case of gynaecological peritonitis [6]. In contrast to France [14], laparotomy was performed on patients with acute generalised peritonitis in this study rather than laparoscopy. Several reasons for this difference are possible. Firstly, it is important to note that the scope of this study was limited to generalised peritonitis, whereas the French study also considered cases of localised peritonitis. Secondly, the low socio-economic status of patients, the paucity of qualified surgeons and the unavailability or lack of laparoscopic equipment in most hospitals in the province. The technique used to manage bowel perforation was bowel resection with terminal anastomosis in 41 cases (40.6%) and ileostomy in 33 cases (32.7%). Many authors have pointed out that there are different proposals for surgery and that the approach used may influence morbidity and mortality [7-10,12]. According to Constantinides *et al*., 2017 [15], the technique used may also depend on the experience of the surgeon. In our series, 75.5% of the procedures were performed by non-surgical doctors. It is uncertain to expect satisfactory results in this context, as suturing in a septic peritoneal cavity is often associated with a high rate of complications (23.1% in our series), such as tissue necrosis with suture loosening, which is the cause of surgical reinterventions (32.4%), and fistula. Ileostomies present challenges for medical professionals for their management. Surgeons and resuscitators must contend with sub-optimal conditions that demand careful consideration. Furthermore, the inadequacy of parenteral and enteral nutrition products in the Democratic Republic of Congo results in malnutrition, which contributes to unfavourable outcomes. A dearth of appropriate ileostomy bags and the challenging task of facilitating the nutrition of patients who suffer significant fluid, ion and dietary mineral loss make this an ongoing issue. Furthermore, the inadequacy of parenteral and enteral nutrition products in the Democratic Republic of Congo results in malnutrition, which contributes to unfavourable outcomes.

This complication rate has been observed in other studies conducted in Rwanda, Lubumbashi in the DRC, and Benin, with rates of 30%, 34.9%, and 18.4% respectively [10,12,16]. Ugumba *et al*., 2018 identified a surgical re-operation rate of 22.3%, and Ndayizeye found a rate of 13% [16]. This rate is indicative of the severity of patients' conditions and the challenging management of AGP in sub-Saharan Africa. The mortality rate was 14.7%. However, this rate of lethality is consistent with that of other studies conducted in sub-Saharan Africa [9,12,17]. A multicentre study [14] conducted in France displays a varying mortality rate based on the location, with a low number of fatalities for appendicular peritonitis patients (1.5%) and a high number of fatalities for patients with colonic peritonitis (23%) and small intestine peritonitis (27%). The mean cost of treating acute generalised peritonitis in urban hospitals was 800.3 USD, with a range of 457 USD to 540 USD and a mean of 377.1 USD, with a range of 350 USD to 544 USD in rural hospitals. This amount is considerably higher than the average income in the Democratic Republic of Congo and may contribute to delayed consultations and higher mortality rates. In Cameroon, a study found that the average total cost of health care was 357,450 F CFA (equivalent to USD 545) [17]. There are some limitations in this study. Firstly, due to its retrospective nature, some clinical data may have been overlooked and the evolution of patients' weight could not be determined. Lastly, the multicentric nature of the study resulted in varying availability and compatibility of patients' medical records and technical platforms across centres. In addition, biological data were mostly absent from the consulted files, which could have provided valuable insight into outcomes and evolving aspects.

## Conclusion

Acute generalised peritonitis is a widespread disease of the young in sub-Saharan Africa. The leading cause is ileal perforation, demanding comprehensive treatment from a proficient medical-surgical team to prevent complications. The investigation of nutritional management is crucial due to the significant impact that nutrition can have on a patient's recovery, infection rates, and overall progression. The forecast remains bleak as the expense of treatment surpasses the median earnings of the populace.

### What is known about this topic

- *In urban Africa, ileal perforation is often main cause of acute generalized peritonitis (AGP), with a mortality rate over to 20%*.

### What this study adds

- *This study showed that the prognosis of peritonitis remains severe and that the cost of treatment is higher than the average income of the population*.
